# Branched‐Chain Amino and Keto Acids Reduce Hepatocyte Lipid Droplet Size and Number via Distinct Proteomic Pathways

**DOI:** 10.1002/pmic.70132

**Published:** 2026-04-20

**Authors:** Jayasimha R. Daddam, Mounica Sura, Edward Kolodziej, Maria Gojcaj, Zheng Zhou

**Affiliations:** ^1^ Department of Animal Science Michigan State University East Lansing USA

**Keywords:** Branched‐chain amino acids, Branched‐chain keto acids, Hepatocytes, Lipid metabolism, Proteomics, TCA cycle

## Abstract

Branched‐chain amino acids (**BCAA**) and their corresponding keto acids (**BCKA**) have been associated with changes in hepatic lipid metabolism and the resulting alterations in intracellular triglyceride concentrations. In this study, we utilized a previously established hepatocyte model to investigate the impact of BCAA and BCKA supplementation on triglyceride accumulation and proteomic responses when exposed to physiologically high levels of fatty acids (**FA**). Confocal imaging revealed that BCAA and BCKA effectively reduced intracellular lipid droplet size and number. Principal component analysis and hierarchical clustering suggest distinct proteomic profiles across treatment groups, with a total of 299 differentially abundant proteins. Gene Ontology and KEGG pathway enrichment analysis revealed distinct effects on hepatic metabolism. Compared to control, BCAA supplementation upregulated proteins in the TCA cycle, oxidative phosphorylation, and thermogenesis, supporting an elevated protein synthesis and energy metabolism. In contrast, BCKA treatment downregulated various proteins in the energy metabolism pathways, including the TCA cycle and oxidative phosphorylation, reflecting a potential feedback mechanism to limit oxidative stress. Increased protein abundance of mitochondrial electron transport chain (ETC) complexes was observed in treated hepatocytes. These results underscore the potential of BCAA and BCKA to regulate hepatic lipid homeostasis in dairy cows when metabolically challenged during early postpartum.

## 1 Introduction

A relationship between circulating branched‐chain amino acid (**BCAA**) levels and hepatic triglyceride (**TG**) concentrations has been observed in both humans and dairy cows [1, 2, 3]. This association suggests that BCAAs may play regulatory roles in lipid metabolism and energy homeostasis beyond their classical functions in protein synthesis and nutrient sensing, underscoring a potential metabolic interplay between BCAA metabolism and hepatic lipid regulation. In dairy cows, circulating BCAA levels are negatively correlated with liver TG accumulation, supporting the observation that BCAA supplementation may offer utility as a nutritional strategy to mitigate hepatic lipidosis [1]. Similarly, in humans, elevated blood BCAA concentrations have also been associated with ballooning and inflammation in patients with nonalcoholic fatty liver disease, likely reflecting increased protein catabolism [4]. Clinically, BCAA supplementation has been utilized to improve the quality of life, nutritional status, and survival of patients suffering from liver diseases, including cirrhosis and hepatic encephalopathy [5, 6]. In these settings, elevated blood BCAA levels help counteract malnutrition and muscle wasting commonly observed in advanced liver disease, thereby improving overall patient outcomes.

**Statement of Significance of the Study**

High hepatic lipid accumulation in dairy cows during early lactation remains a critical metabolic challenge, leading to fatty liver, weak immune function, and reduced productivity. Although branched‐chain amino acids (BCAA) and their corresponding branched‐chain keto acids (BCKAs) are associated with regulating energy and lipid metabolism, their mechanistic roles in the liver are not clearly defined. This study provides the first comprehensive, side‐by‐side evaluation of the effects of BCAA and BCKA on hepatocyte lipid droplet dynamics and global proteomic remodeling in physiologically high fatty acid conditions. By integrating high‐resolution confocal imaging with in‐depth quantitative proteomics, we demonstrate that both BCAA and BCKA effectively reduce the number and volume of lipid droplets, while activating fundamentally different metabolic programs. BCAA supplementation enhances amino acid metabolism, mitochondrial oxidative phosphorylation, and protein synthetic capacity, while BCKA suppresses energy‐intensive pathways, suggesting protective modulation against oxidative stress. These distinct proteomic signatures provide mechanistic insights that help explain contrasts with the in vivo observations in dairy cows. The findings advance our understanding of BCAA/BCKA‐regulated hepatic metabolism and identify different metabolic pathways that aim to reduce early‐lactating fatty liver. This work provides a valuable framework for developing treatment strategies to improve the hepatocyte mechanism to reduce fatty acid accumulation.

Despite the associations between circulating BCAA concentrations and hepatic TG accumulation, direct hepatic uptake and catabolism of BCAA are relatively minimal. This phenomenon is primarily due to the inherently low activity of branched‐chain amino acid aminotransferase (BCAT) in hepatocytes, the enzyme responsible for the initial transamination of BCAA to their respective branched‐chain keto acids (**BCKA**) [7, 8, 9]. Consequently, skeletal muscle and adipose tissue serve as major sites for BCAA catabolism [10, 11]. Although the initial step of BCAA catabolism occurs predominantly in extrahepatic tissues, these tissues possess limited capacity to oxidize further the resulting BCKA [7]. As a result, up to 50% of the BCKA generated are subsequently catabolized in the liver [7]. This hepatic processing of BCKA may contribute to the observed inverse relationship between circulating BCAA levels and hepatic TG concentrations.

Accordingly, the extent of TG reduction in hepatocytes when exposed to physiologically high fatty acids (**FA**) may depend on the amount of BCKA shuttling to the liver. Supporting this hypothesis, recent results from our in vivo study observed distinct responses following supplementation of equal molar amounts of BCAA or their corresponding BCKA in dairy cows during early lactation. Specifically, BCKA supplementation reduced liver TG, whereas BCAA supplementation did not influence hepatic TG despite a significant increase in milk production being observed. These contrasting observations imply distinct metabolic roles and pathways activated by BCAA and BCKA, warranting further exploration into their differential mechanisms of action in hepatic metabolism.

Building on these observations, our recent in vitro experiments demonstrated that BCAA or BCKA supplementation significantly decreased TG accumulation in hepatocytes exposed to physiologically high FA levels, as determined by flow cytometry‐based quantification of intracellular total lipids [12]. However, it remains unclear whether this reduction in TG is due to changes in lipid droplet size, number, or both. Hence, the objectives of this study were to assess the impact of BCAA and BCKA supplementation on intracellular lipid droplet size and number using confocal microscopy and elucidate the mechanisms underlying their differential effects on hepatic metabolism. We hypothesize that BCAA and BCKA supplementation mitigate intracellular lipid accumulation when exposed to physiologically high level of FA by reducing lipid droplet size and number, which are associated with distinct alterations in hepatic protein abundance and metabolic pathways.

## 2 Materials and Methods

### 2.1 Isolation and Culture of Primary Hepatocytes

Primary hepatocytes were isolated from mid‐lactation dairy cows as previously described [13]. These hepatocytes express hepatocyte‐specific markers and exhibit functional characteristics, including albumin and urea synthesis [13]. Cell viability exceeded 99%, as assessed by trypan blue exclusion using an automated cell counter (Countess II FL, Thermo Fisher Scientific, Waltham, MA) prior to plating. Hepatocytes were seeded in 6‐well culture plates at a density of 4 × 10^5^ cells per well in Dulbecco's Modified Eagle Medium (DMEM) supplemented with 10% fetal bovine serum, 1 nM glucagon, 10 nM dexamethasone, 10 ng/mL epidermal growth factor, 10 nM insulin, and 1% penicillin/streptomycin [14]. Cells were maintained until ∼80% confluence was achieved, at which point they were washed three times with phosphate‐buffered saline prior to treatment.

To closely mimic the in vivo hepatic environment during early lactation characterized by elevated circulating FA and low amino acid (**AA**) concentrations, a customized serum‐free medium was prepared and used as the physiological control (CON). In this medium, concentrations of all 20 AA, volatile fatty acids (VFAs), and FA (1 mM consisting 23.0% C16:0, 23.0% C18:0, 19.7% C18:1, 29.1% C18:2, and 5.3% C20:4), glucose, choline, insulin, and albumin are equivalent to plasma levels observed in control cows on day 4 postpartum as established previously [15, 16].

### 2.2 BCAA and BCKA Treatments

Data from a previous study [17] indicated that cows with low hepatic TG concentrations on day 7 postpartum had, on average, 33% higher circulating BCAA levels on day 4 postpartum compared to cows with high liver TG [18]. Since BCAA are primarily transaminated to BCKA in extrahepatic tissues, and BCKA are subsequently oxidized in the liver [19]. Treatments were designed to mimic this physiological difference. The treatment groups were as follows: 1) CON: physiological control using the customized serum‐free medium; 2) BCAA: CON supplemented with 33% additional BCAA (valine, leucine, isoleucine) relative to their circulating concentrations on day 4 postpartum; 3) BCKA: CON supplemented with 33% additional BCKA corresponding to the plasma concentrations of their parent BCAA on day 4 postpartum. Each treatment group included six biological replicates.

Previous data showed that plasma concentrations of valine, leucine, and isoleucine increased by 79%, 60%, and 84%, respectively, between days 1 and 7 postpartum [20]. Therefore, mid‐week (day 4) concentrations were selected to represent average BCAA levels during the early lactation period [20]. Cells were incubated under these treatment conditions for 72 h unless otherwise specified.

### 2.3 Confocal Microscopy and Image Analysis

To prepare samples for imaging, sterile glass microscope slides were placed into each well of a standard 6‐well plate, and cells were seeded directly onto the slides. After experimental treatments, cells were fixed in 4% paraformaldehyde at room temperature for 15 min, followed by three PBS washes. The fixed cells were then stained at room temperature for 30 min with Hoechst 33342 (1 µg/mL) to visualize nuclei and BODIPY 493/503 (200 nM) to label neutral lipid droplets (**LD**).

Sequential laser excitation was used to reduce spectral bleed‐through: Hoechst was excited at 350 nm with a 30 mW diode laser and detected using a 461 nm short‐pass filter, while BODIPY 493/503 fluorescence was collected using a 500–550 nm band‐pass filter.

Images were captured using a Zeiss PALM MicroBeam IV system with confocal capabilities. Super‐resolution images were obtained using a 60× oil immersion apochromat objective equipped with an Airyscan detector. For general observations and standard‐resolution imaging, a 40× air objective was used. Z‐stacks (0.3‐0.5 µm spacing) were collected for each sample and processed into maximum intensity projections for analysis [21].

Image analysis was performed using Fiji (ImageJ, NIH). Individual hepatocytes were manually outlined using Hoechst‐stained nuclei as a guide. LD were identified from BODIPY signal using an automated global threshold, using the “Analyze Particles” function after applying a size filter to exclude objects smaller than 10 pixels.

### 2.4 Protein Extraction and Digestion for LC‐MS

Aliquots containing 10 µg of total protein from each sample were digested using S‐Trap micro spin columns (Protifi, Huntington, NY) according to the manufacturer's protocol. Proteins were reduced, alkylated, and digested with sequencing‐grade modified trypsin (Promega) at a 1:50 (w/w) enzyme‐to‐substrate ratio overnight at 37°C. Peptides were eluted, dried by vacuum centrifugation, and reconstituted in 20 µL of 2% acetonitrile, 0.1% trifluoroacetic acid. Peptide concentrations were determined using the Pierce Quantitative Colorimetric Peptide Assay (Thermo Scientific, #23275) [22].

### 2.5 LC–MS/MS Analysis

Protein samples (100 µg) were precipitated with acetone at ‐20°C overnight. Protein pellets were resuspended in 270 µL of 100 mM Tris‐HCl (pH 8.5) supplemented with 4% (w/v) sodium deoxycholate (SDC). Samples were reduced and alkylated by adding TCEP and chloroacetamide at 10 and 40 mM, respectively, and incubated for 5 min at 45°C with shaking at 2000 rpm in an Eppendorf ThermoMixer C. Trypsin, in 50 mM ammonium bicarbonate, was added at a 1:100 ratio (wt/wt), and the mixture was incubated at 37°C overnight with shaking at 1500 rpm in the Thermomixer. The final volume of each digest was ∼300 µL. After digestion, SDC was removed by phase extraction. The peptide samples were acidified to 1% TFA and subjected to C18 solid phase clean up using StageTips to remove salts eluted with 80% acetonitrile/0.1% formic acid, vacuum‐dried, and reconstituted in 0.1% formic acid for LC–MS/MS injection. Peptide injection of 5 uL was automatically made using a Thermo (www.thermo.com) EASYnLC 1200 onto a Thermo Acclaim PepMap RSLC 0.1 mm x 20 mm C18 trapping column and washed for ∼5 min with buffer A. Bound peptides were then eluted over 65 min onto a Thermo Acclaim PepMap RSLC 0.075 mm x 500 mm resolving column with a linear gradient of 5%B to 28%B in 54 min. After the gradient, the column was washed with 90%B for 10 min (Buffer A = 99.9% Water/0.1% Formic Acid, Buffer B = 80% Acetonitrile/0.1% Formic Acid/19.9% Water) at a constant flow rate of 300 nl/min. Column temperature was maintained at a constant temperature of 50°C using and integrated column oven (PRSO‐V2, Sonation GmbH, Biberach, Germany). Eluted peptides were sprayed into a Thermo Scientific Q‐Exactive HF‐X mass spectrometer (www.thermo.com) using a FlexSpray spray ion source. MS data were acquired in data‐dependent acquisition mode (DDA). Full MS scans were collected at 45,000 resolution (m/z 200) over m/z 350–1500 (AGC: 3 × 10^6^; max IT: 45 ms). The top 15 most intense precursors were fragmented by HCD (NCE 28) and analyzed at 7,500 resolution (AGC: 1 × 10^5^; max IT: 22 ms; isolation width: 1.6 m/z; dynamic exclusion: 30 s).

### 2.6 Data Analysis

Raw MS files were processed using MaxQuant v2.x with the integrated Andromeda search engine to generate peak lists. Spectra were searched against a custom FASTA database composed of *Bos taurus* reference proteins (UniProt, downloaded March 13, 2025) and common contaminants (cRAP, GPM.org) [23]. Search parameters included strict trypsin specificity, up to two missed cleavages, precursor mass tolerance of ±10 ppm, fragment mass tolerance of 0.02 Da, fixed carbamidomethylation of cysteine, peptide tolerance of ± 4.5 ppm and variable oxidation of methionine and N‐terminal acetylation. False discovery rates (FDR) were calculated using a decoy database strategy.

The MaxQuant output was then analyzed using Scaffold, v5.3.1 (www.proteomesoftware.com) to probabilistically validate protein identifications. False discovery rate (FDR) confidence filter are considered true for 1% for peptides, 1% for proteins, and 1% for modification sites using target–decoy estimation. Only proteins supported by at least two unique peptides were quantified [24]. Label‐free quantification (LFQ) was performed with MaxLFQ algorithm by enabling “Match Between Runs” (0.7‐min match window; 20‐min alignment window), and intensity values were extracted from the *proteinGroups.txt* output.

### 2.7 Data Processing and Statistical Analysis

Data processing and protein identification were performed in Scaffold, v5.3.1 (www.proteomesoftware.com). Proteins annotated as contaminants were removed. LFQ intensities were log_2_‐transformed, and proteins were retained only if quantified in more than 4 out of 6 biological replicates per group. Missing values were imputed using a left‐shifted normal distribution (width = 0.3; downshift = 1.8). Differentially abundant proteins were identified by one‐way ANOVA with Benjamini–Hochberg correction (FDR < 0.05) and log_2 µ_fold change ≥ 0.58.

### 2.8 Principal Component Analysis (PCA)

Normalized spectral counts from Scaffold were standardized across samples for principal component analysis (PCA). PCA was conducted in R v4.1.2 using the ggplot2 package. The first two principal components were plotted to visualize sample variance and identify clustering or outliers.

### 2.9 Hierarchical Clustering

Hierarchical clustering was performed using log2‐transformed and z‐score standardized spectral counts. Distance matrices were computed using Euclidean distance and clustered with Ward's linkage method. Heatmaps and dendrograms were generated to visualize expression profiles and clustering of proteins across samples (Figure S1).

### 2.10 Volcano Plot Analysis

Differential protein expression was assessed using log2 fold change (logFC) and –log10‐transformed p‐values derived from ANOVA. Volcano plots were generated using ggplot2 and ggrepel in R v4.1.2. Significantly differentially abundant proteins were defined by logFC > 1.25 and *p* < 0.05 and highlighted as follows: red (upregulated), blue (downregulated), and gray (nonsignificant).

### 2.11 Gene Ontology (GO) and KEGG Pathway Enrichment Analysis

Differentially abundant proteins (DAP) for each treatment comparison were analyzed for GO term enrichment (biological process, molecular function, and cellular component) using STRING (v12.0). Statistical enrichment was determined using a hypergeometric test with *FDR* < 0.05. Results were visualized with bar plots or network diagrams using the SR Plot Server (26). KEGG pathway enrichment was performed in STRING using DAP (*FDR* < 0.05, logFC > 1.3). Upregulated and downregulated protein lists were analyzed separately, and significantly enriched pathways (*FDR* < 0.05) were ranked by enrichment strength [25].

### 2.12 Western Blot Analysis

Hepatocytes were lysed in NP‐40 buffer supplemented with protease inhibitors [26]. Cell debris was removed by centrifugation, and protein concentration was determined using the bicinchoninic acid (BCA) assay [27, 28]. Proteins were separated using sodium dodecyl sulfate‐polyacrylamide gel electrophoresis (SDS‐PAGE) and transferred to PVDF membranes using a semi‐dry or wet transfer apparatus [29]. Membranes were blocked with 5% nonfat milk and incubated with OxPhos Rodent WB Antibody Cocktail (Cat#: 45–8099, Invitrogen) or β‐Actin (13E5) Rabbit mAb HRP‐conjugate (Cat#: 5125S, Cell Signaling Technology). HRP‐linked anti‐mouse or anti‐rabbit secondary antibodies (Cat#: 7076S, Cell Signaling Technology; Cat#: 406421, BioLegend) were applied accordingly [30]. Bands were detected via enhanced chemiluminescence and imaged using a digital imaging system. Band intensity was quantified using Image Lab software [31].

### 2.13 Statistical Analysis

The effect of treatment on LD number, area, and mitochondrial complexes abundance were determined using the PROC GLIMMIX procedure of SAS 9.4. Data were reported as least squares means with associated standard errors. Statistical differences were declared significant at *FDR* ≤ 0.05 and tendencies at 0.05< *FDR* ≤ 0.10.

## 3 Results

### 3.1 BCAA and BCKA Reduced Intracellular TG Accumulation in Hepatocytes

**FIGURE 1:**
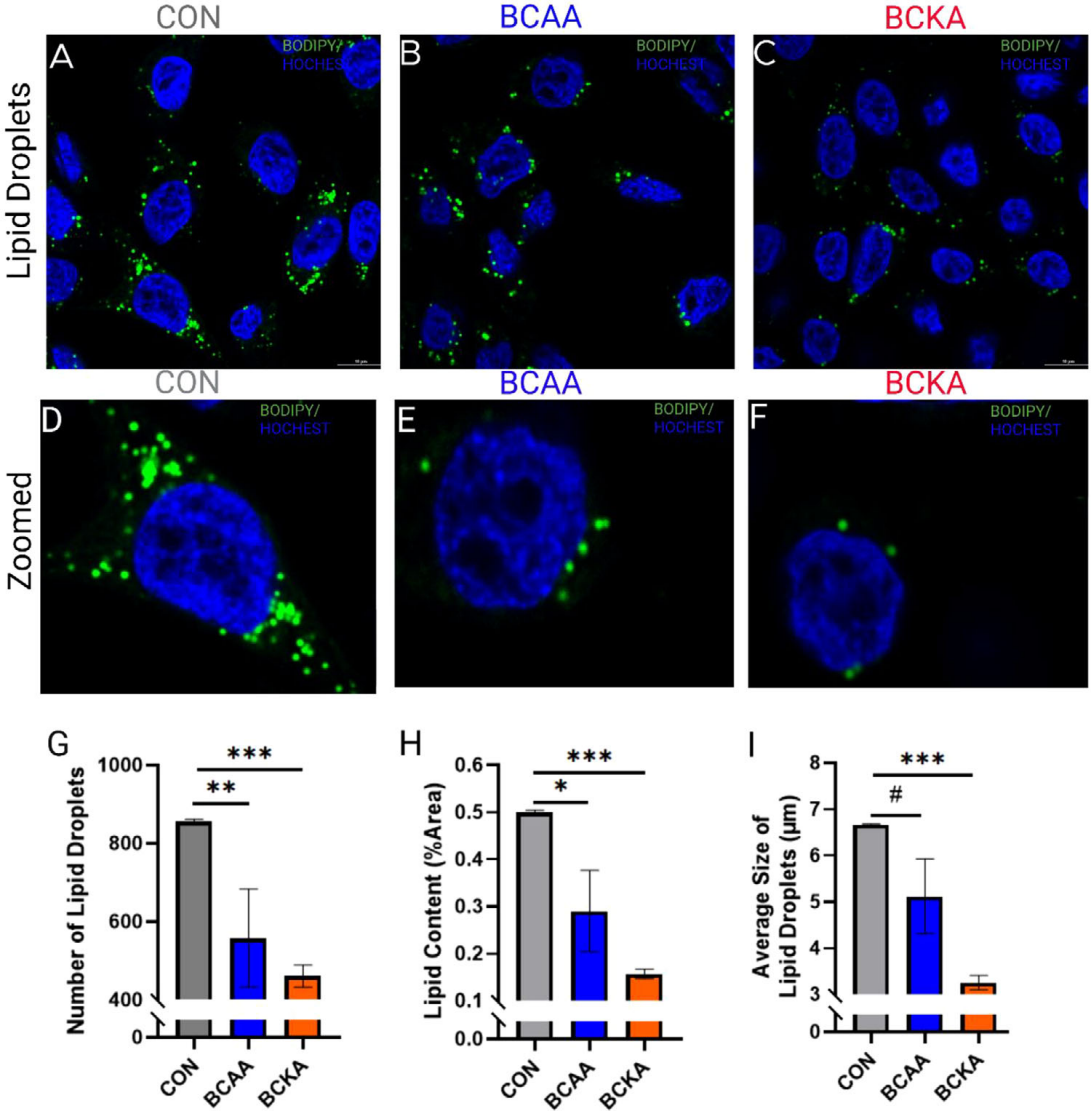
BCAA and BCKA treatments reduce lipid accumulation in hepatocytes. (A‐C) Representative confocal microscopy images of lipid droplets (green) stained with BODIPY 493/503 in CON (1 mM FA cocktail mimicking circulating FAs), BCAA‐treated (CON + 33% additional BCAA of corresponding circulating BCAA concentration), and BCKA‐treated (CON + 33% BCKA of corresponding circulating BCAA concentration) hepatocytes (scale bar: 10 µm).(D‐F) High‐resolution images showing lipid droplets (green, BODIPY 493/503), and nuclei (blue, Hoechst) under corresponding treatments. (G‐H) Quantification of LD number (G) and total LD area (H) per cell (n = 17–23 hepatocytes/group, 6 independent experiments). (I) Frequency distribution of LD sizes (n = 350–450 LD/group). Data: mean ± SEM; ***p < 0.01, *p < 0.05, ^#^ *p* < 0.1.

Flow cytometry‐based quantification previously demonstrated that both BCAA and their corresponding BCKA reduced TG concentration in hepatocytes when exposed to physiological high levels of FA [12]. To confirm and visualize these results, LD number, average LD volume, and lipid content as % area were determined using confocal microscopy (Figure 1). Exposure to physiologically high levels of FA for 72 h resulted in the accumulation of intracellular TG in primary bovine hepatocytes (Figure 1A,D, control (CON)). Compared to CON (856 ± 6, average ± SEM), BCAA supplementation decreased the number of LD by BCAA by 30% (558 ± 125, *p* < 0.001; Figure 1B,E,G). Consequently, the total lipid content, calculated as the percentage area, was significantly lower in BCAA supplemented hepatocytes compared to CON (BCAA: 0.20% ± 0.08 vs. CON: 0.40% ± 0.01, *p* = 0.035; Figure 1H). Similarly, the size of LD (5.11 ± 0.8 µm, diameter) was also lower in response to BCAA supplementation compared to CON (6.6 ± 0.01 µm; *p* = 0.084; Figure 1I). Similarly, BCKA supplementation also significantly reduced LD number (461 ± 28, *p* < 0.001), mean LD volume (3.2 ± 0.15 µm, *p* < 0.001), and total lipid content (0.15% ± 0.01, *p* < 0.001; Figure 1C,F) compared to CON. These results support that BCAA and BCKA both reduced FA‐induced TG accumulation in hepatocytes.

### 3.2 BCAA and BCKA Treatments Induce Distinct Hepatic Proteomic Profiles

**FIGURE 2:**
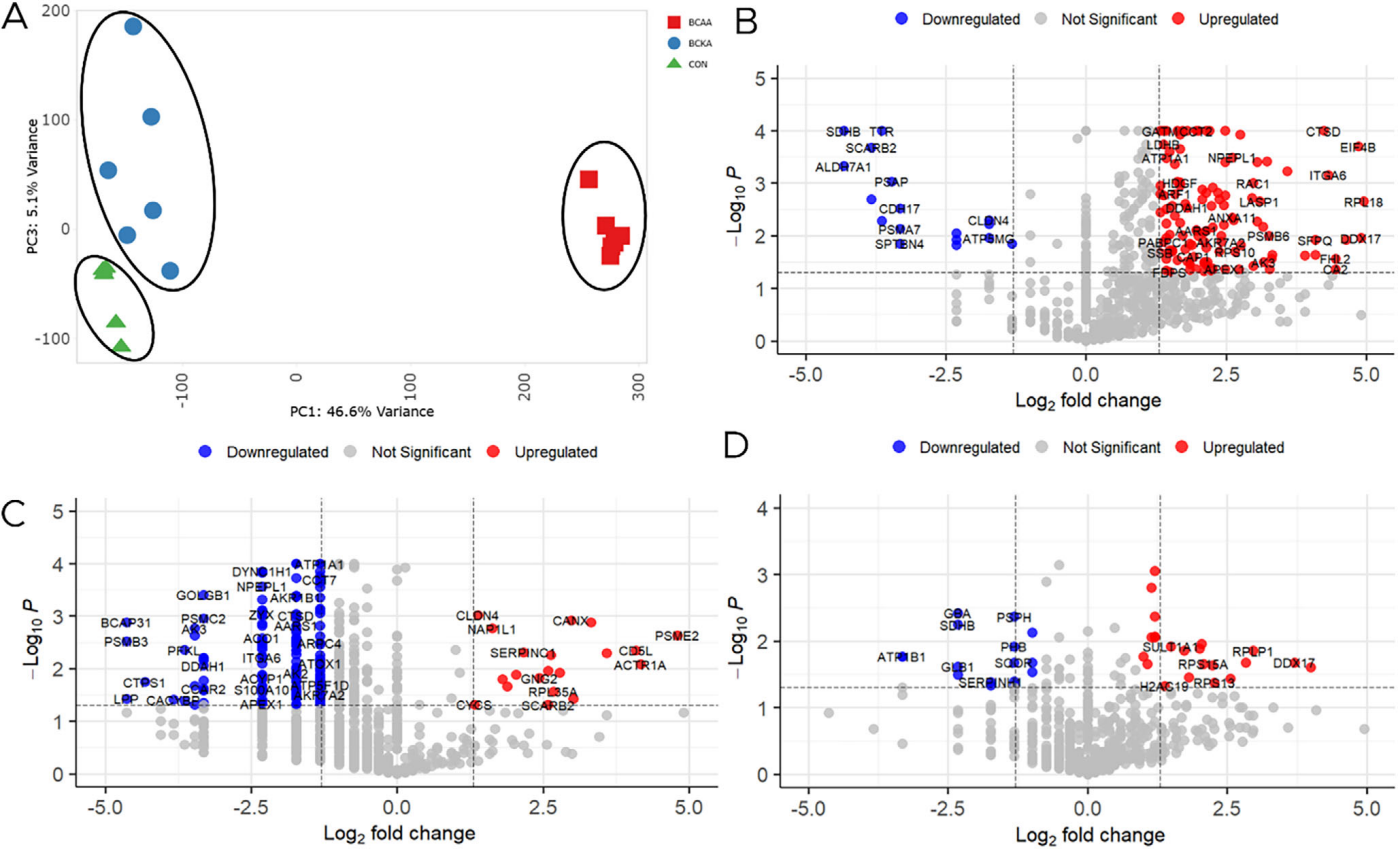
Multivariate and differential abundance analyses of hepatocyte proteomes across treatment groups. (A) Principal component analysis (PCA) of differentially abundant proteins demonstrates clear separation among BCAA (red squares), BCKA (blue circles), and CON (green triangles) treatment groups, explaining 51.7% of total variance. (B–D) Volcano plots illustrate DAPs for each pairwise comparison: BCAA vs. CON (B), BCAA vs. BCKA (C), and BCKA vs. CON (D). Significantly upregulated proteins are shown in red, downregulated proteins in blue. The x‐axis represents log_2_ fold change, and the y‐axis indicates –log_10_ *p*‐value. DAPs were identified using a threshold of fold change ≥1.25 and *FDR* < 0.05.

A total of 1,547 proteins were identified in bovine hepatocyte samples. Of these, 988 unique proteins were consistently quantified across all samples. The dataset comprised 126,936 raw spectral counts, corresponding to 40,370 unique spectra and 36,242 unique peptides. These peptides were mapped to the 988 quantified proteins. Differential abundance analysis across the CON, BCAA, and BCKA treatment groups identified 299 differentially abundant proteins (DAP) based on a *FDR* < 0.05. The complete list of DAP is provided in Supplemental Table S1. PCA analysis revealed that biological replicates from each treatment group clustered distantly and were clearly separated from other treatments (Figure 2A). As visualized in the volcano plots (Figure 2B,D), pairwise comparisons of BCAA vs. CON, BCKA vs. CON, and BCAA vs. BCKA also revealed numerous DAP, supporting a distinct proteomic profile between treatments. Specifically, compared to CON, BCAA treatment upregulated 197 proteins and downregulated 17 proteins, respectively (Figure 2B). Similarly, 17 proteins were significantly upregulated and 203 proteins downregulated in BCAA compared with BCKA (Figure 2C). In contrast, when compared to CON, BCKA upregulated 14 proteins and downregulated 11 proteins (Figure 2D).

### 3.3 Gene Ontology (GO) Analysis Highlights AA Metabolism, Protein Folding, and Mitochondrial Functions

**FIGURE 3:**
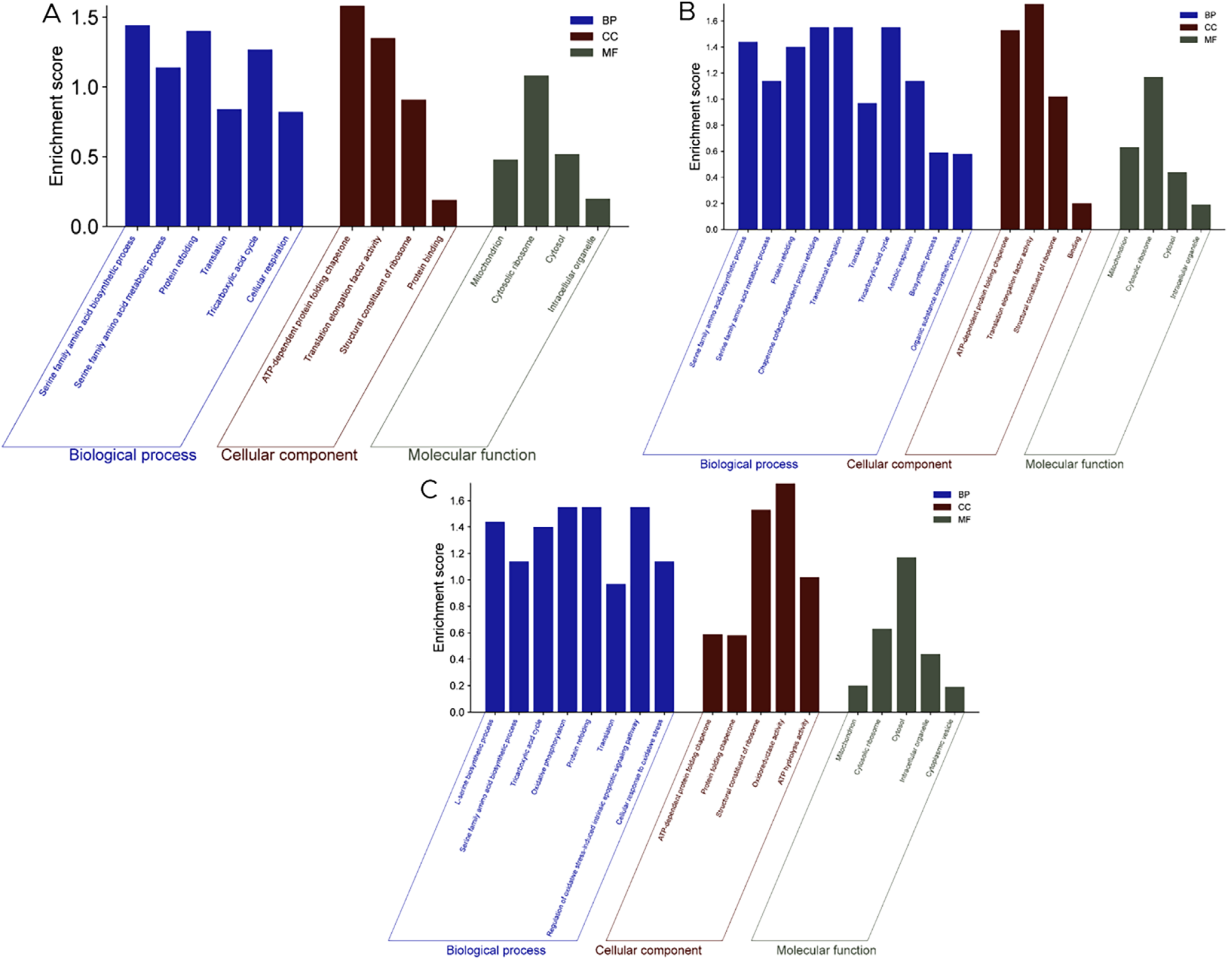
Gene Ontology (GO) enrichment analysis of differentially abundant proteins between treatment groups. Comparisons include BCAA vs. CON (A), BCKA vs. CON (B), and BCAA vs. BCKA (C). GO terms were categorized into Biological Process (BP), Molecular Function (MF), and Cellular Component (CC). Bar plots display significantly enriched GO terms (*FDR* < 0.05) for each category, with enrichment scores plotted on the y‐axis.

Gene Ontology (GO) enrichment analysis of the differentially abundant proteins (DAP) revealed significant enrichment across all three GO categories—Biological Process (BP), Cellular Component (CC), and Molecular Function (MF)—for each treatment comparison. Representative terms for each comparison were presented in Figure 3. In the BCAA versus CON comparison, GO enrichment analysis revealed significant enrichment in biological processes including AA metabolism and biosynthesis (GO:0009070, GO:0009069), protein folding and translation (GO:0042026, GO:0006412), and cellular respiration (GO:0006099, GO:0045333). Molecular functions included protein folding and translation regulation (e.g., GO:0140662, GO:0003746), structural roles (GO:0003735), and binding activities (GO:0005515). Cellular components highlighted mitochondrial and ribosomal structures (GO:0005739, GO:0022626), cytosolic components (GO:0005829), and intracellular organelles (GO:0043229) (Figure 3A). In the BCKA versus CON comparison, key biological processes included AA metabolism and biosynthesis (GO:0009070, GO:0009069), protein folding and refolding (GO:0042026, GO:0051085), translation (GO:0006414, GO:0006412), cellular respiration (GO:0006099, GO:0009060), and biosynthetic processes (GO:0009058, GO:1901576). Molecular functions were enriched for protein folding and translation regulation (GO:0140662, GO:0003746), structural roles (GO:0003735), and binding activities (GO:0005488). Cellular components highlighted mitochondrial and ribosomal structures (GO:0005739, GO:0022626), cytosolic components (GO:0005829), and intracellular organelles (GO:0043229) (Figure 3B). When comparing BCAA versus BCKA, key GO terms include AA metabolism and biosynthesis (GO:0006564, GO:0009070), energy production pathways such as the tricarboxylic acid cycle and oxidative phosphorylation (GO:0006099, GO:0006119), and protein folding and translation regulation (GO:0042026, GO:0006412). Additionally, processes involved in apoptosis and cellular stress responses (GO:1902175, GO:0034599) were also enriched. Molecular functions were dominated by protein folding and chaperone activities (GO:0140662, GO:0044183), structural roles (GO:0003735), and catalytic activities, including oxidoreductase and ATPase functions (GO:0016491, GO:0016887). Cellular components were primarily associated with mitochondrial and ribosomal structures (GO:0005739, GO:0022626), cytosolic and intracellular organelles (GO:0005829, GO:0043229), and membrane‐bound vesicles (GO:0031410) (Figure 3C).

### 3.4 KEGG Pathway Analysis Reveals Distinct Metabolic Responses to BCAA and BCKA Treatments

**FIGURE 4:**
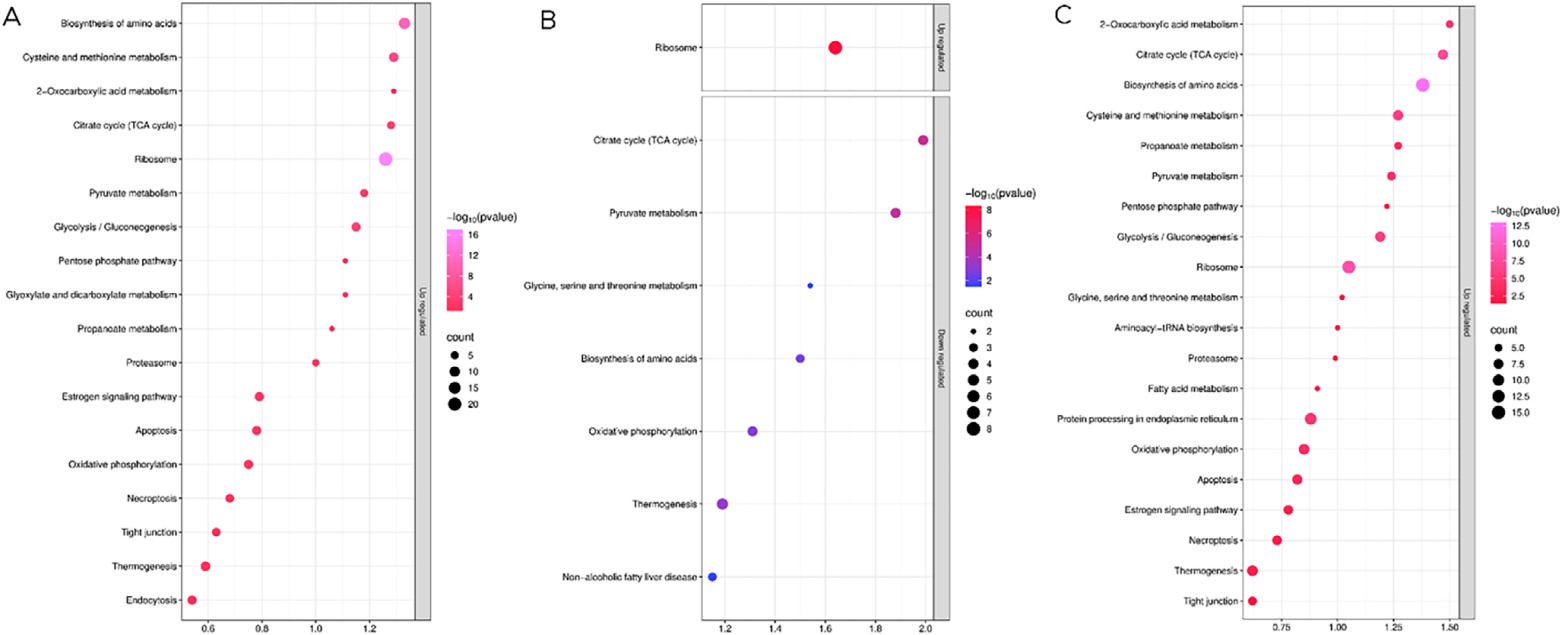
KEGG pathway enrichment analysis of differentially abundant proteins in BCAA‐ and BCKA‐treated hepatocytes compared to control. (A‐C) Top significantly enriched KEGG pathways (FDR < 0.05) identified in BCAA vs. CON (A), BCKA vs. CON (B), and BCAA vs. BCKA (C) comparisons. The y‐axis lists enriched pathways, and the x‐axis displays enrichment scores. Dot size represents the number of proteins contributing to each pathway, while dot color indicates statistical significance (–log_10_ *p*‐value).

Top pathways enriched by up‐ or down‐regulated proteins from each of the treatment comparisons are presented in Figure 4. KEGG pathway enrichment analysis revealed distinct patterns of biological activity associated with the DAP. Compared to CON, up‐regulated DAP by BCAA treatment significantly (*FDR* < 0.05) enriched 20 pathways regulating protein and AA metabolism, energy metabolism, apoptosis, and endocytosis (Figure 4A). Among the up‐regulated pathways, the biosynthesis of AA pathway demonstrated the most significant enrichment (*FDR* < 0.01), with 13 out of 67 pathway‐associated proteins showing greater abundance in response to additional BCAA compared to CON. Similarly, various other pathways regulating AA and protein metabolism were also up‐regulated in response to BCAA treatment compared to CON, including cysteine and methionine metabolism (8/46 proteins up‐regulated), protein processing in the endoplasmic reticulum (13/157 proteins up‐regulated), proteasome (4/44 proteins up‐regulated), and ribosome (22/134 proteins up‐regulated). Various pathways regulating energy metabolism were also up‐regulated by BCAA treatment compared to CON, including the TCA cycle (5/29 proteins up‐regulated), glycolysis/gluconeogenesis (7/55 proteins up‐regulated), and oxidative phosphorylation (7/137 proteins up‐regulated). Down‐regulated proteins did not result in any significantly (*FDR* < 0.05) enriched pathways from the BCAA versus CON comparison. In contrast, compared to CON, up‐regulated proteins from BCKA treatment only enriched Ribosome pathway (8/134 proteins up‐regulated). However, down‐regulated proteins enriched various pathways regulating AA and energy metabolism, including TCA cycle (4/29 proteins down‐regulated), oxidative phosphorylation (4/137 proteins down‐regulated), thermogenesis (5/130 proteins down‐regulated), pyruvate metabolism (4/37 proteins down‐regulated), glycine, serine, and threonine metabolism (2/41 proteins down‐regulated), and biosynthesis of AA (3/67 proteins down‐regulated) (Figure 4B). Consequently, these AA and energy metabolism pathways were significantly enriched by up‐regulated proteins from the BCAA versus BCKA comparison (Figure 4C).

### 3.5 BCAA and BCKA Modulate Abundance of Specific Electron Transport Chain Complex Proteins

**FIGURE 5:**
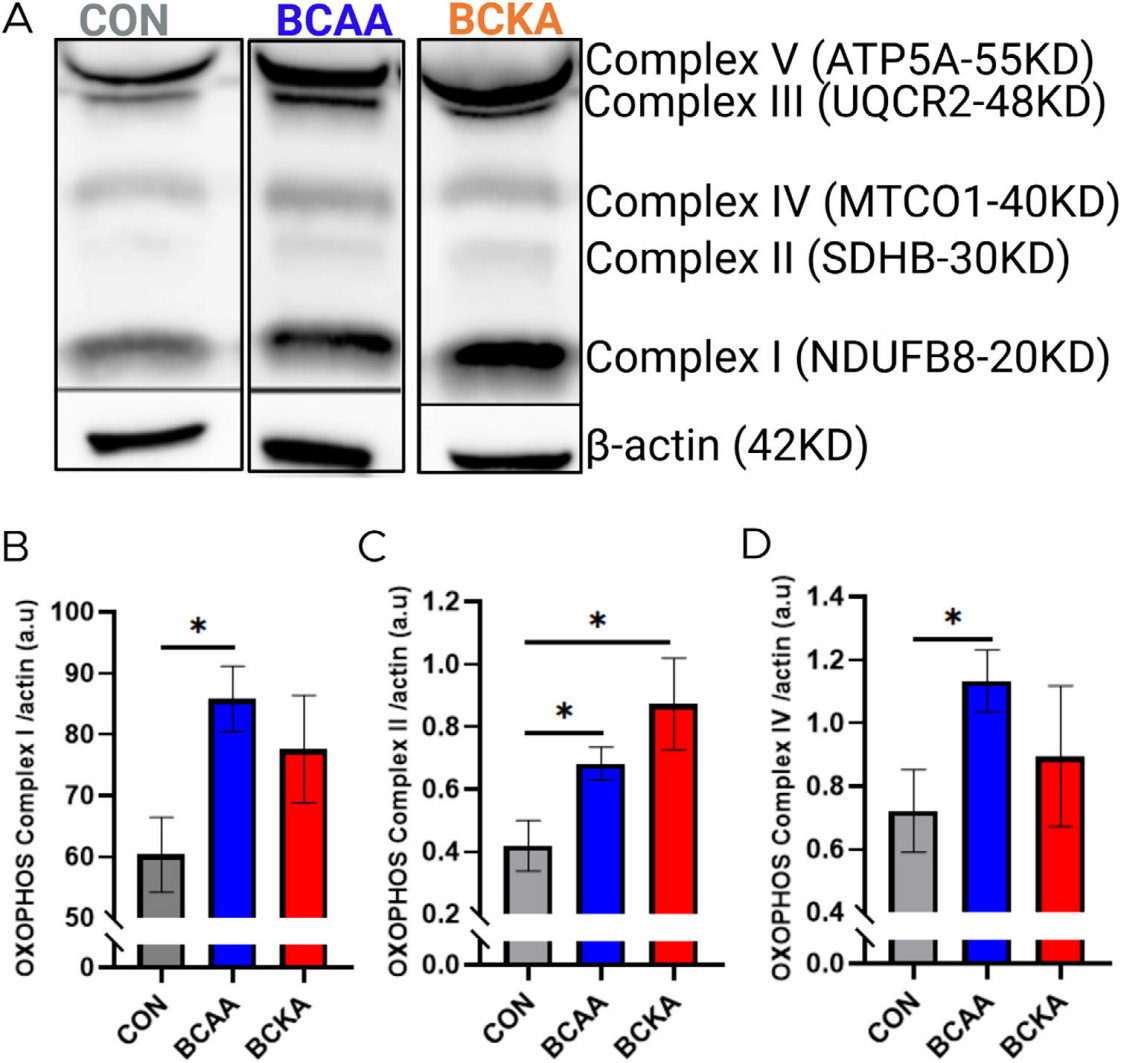
Protein abundance of mitochondrial electron transport chain (ETC) complexes in hepatocytes. (A) Representative Western blots showing expression of ETC complex subunits ATP5A (Complex V), NDUFB8 (Complex I), UQCRC2 (Complex III), MTCO1 (Complex IV), and SDHB (Complex II) in control (CON), BCAA‐, and BCKA‐treated samples. β‐actin was used as the loading control. (B) Quantification of NDUFB8, SDHB, and MTCO1 protein abundance normalized to β‐actin. * indicates *p*‐value < 0.05.

The protein abundance of electron transport chain (ETC) complexes (I‐V), associated with oxidative phosphorylation, was studied in hepatocytes, comparing BCAA/BCKA to CON samples. β‐actin served as the internal control for quantifying the abundance in six samples of each type (Figure 5). Notably, the average abundance of Complex I (NDUFB8‐20KD) was significantly higher in BCAA samples compared to CON samples (*p* = 0.010; Figure 5A), and Complex II (SDHB‐30KD) also showed a significant increase in both BCAA (*p* = 0.021) and BCKA (*p* = 0.022) treatments compared to CON samples (Figure 5B). Complex IV (MTCO1‐40KD) showed significantly higher abundance in BCAA but not in BCKA compared to CON samples (*p* = 0.030; Figure 5C). However, no significant change was observed for other complexes between BCAA/BCKA and CON samples (Figure S2).

## 4 Discussion

Previous findings from our group have shown that BCAA and BCKA supplementation share some similar effects on bovine hepatic metabolism but also exhibit important distinctions, both in vitro and in vivo. In vitro, both BCAA and BCKA supplementation reduced intracellular TG concentrations by 32% and 40%, respectively, in primary bovine hepatocytes exposed to FA. These reductions were accompanied by increased expression of FA oxidation genes, decreased oxidative stress, and reduced apoptosis [10, 11]. In contrast, in vivo studies revealed that only BCKA infusion significantly reduced liver TG content (4.77% vs. 6.60% in controls) in early postpartum cows, while BCAA supplementation did not alter hepatic TG but improved milk production [7]. Together, these findings suggest that although supplemented at equal molar amount, BCAA and BCKA affect different aspects of hepatic metabolism in vitro and in vivo, supporting further investigation of the molecular mechanisms involved.

### 4.1 BCAA and BCKA Reduce Hepatic Lipid Accumulation via Lipid Droplet Remodeling

Building on our prior observations, the current study further confirms that both BCAA and BCKA supplementation reduce intracellular TG accumulation in bovine hepatocytes exposed to physiologically high levels of FA. Using confocal microscopy, we demonstrate that this reduction in TG content is accompanied by a significant decrease in LD number and a modest reduction in LD size. These morphological changes in LD characteristics likely reflect altered lipid handling within hepatocytes, potentially involving enhanced lipolysis, suppressed TG synthesis, or both. Importantly, the similarity in the extent of their effects might be explained by the fact that BCAA and BCKA share a common downstream metabolic fate once deaminated. However, given that BCAA catabolism is initiated predominantly in extrahepatic tissues (i.e., skeletal muscle and adipose tissue) while BCKA are primarily metabolized in the liver, the site of entry into catabolic pathways may influence the cellular responses observed. The changes in LD size and number serve as functional readouts of intracellular lipid homeostasis and offer a morphological basis for the improved oxidative and anti‐apoptotic profiles we previously reported [10, 11] and further highlight the need to understand the broader metabolic adaptations—encompassing amino acid metabolism, fatty acid catabolism, and related pathways—that may contribute to or result from the observed lipid remodeling.

### 4.2 Differential Impact on AA Metabolism

In vivo, BCAA and their corresponding BCKA are interconvertible through reversible transamination reactions [32]. In addition to the consequent increase in BCAA availability to the hepatocytes receiving BCAA and BCKA treatments, the availability of other AA is also expected to increase as additional BCAA and BCKA may spare other AA from entering the TCA cycle for energy. With an equal molar amount supplemented in the BCAA and BCKA treatments in this study, we expect to observe a similar impact on AA metabolism in hepatocytes. As expected, GO enrichment analysis revealed that BCAA supplementation significantly upregulated pathways related to AA metabolism and biosynthesis (GO:0009070, GO:0009069). KEGG pathway enrichment analysis also revealed that BCAA supplementation significantly upregulated pathways related to AA metabolism and biosynthesis, including cysteine and methionine metabolism and biosynthesis of AA, suggesting a direct link between BCAA supplementation and increased AA availability and utilization. In contrast, BCKA supplementation downregulated glycine, serine, and threonine metabolism and biosynthesis of AA pathways, suggesting a distinct metabolic shift.

Such differential responses are likely due to the low branched‐chain AA aminotransferase (BCAT) activity and high branched‐chain keto acid dehydrogenase (BCKD) activity in hepatocytes [32]. With BCAA deamination to corresponding BCKA by BCAT as the first step of BCAA catabolism, low BCAT activity in hepatocytes is the main reason BCAA largely escape first‐pass hepatic catabolism to be catabolized in extra‐hepatic tissues (e.g., skeletal muscle, adipose tissue) [7, 20]. Hence, instead of being degraded to BCKA, the additional BCAA available to hepatocytes from BCAA supplementation are likely directed toward protein synthesis. Consequently, utilization of other AA will increase, hence the up‐regulation of biosynthesis of AA pathway and enzymes in the cysteine and methionine metabolism pathway to support methionine regeneration through the methionine cycle (e.g., phosphoglycerate dehydrogenase (PHGDH), phosphoserine aminotransferase 1 (PSAT1)). Conversely, with high BCKD activity in hepatocytes, the additional BCKA available to hepatocytes from BCKA supplementation is likely directed toward the energy production from the TCA cycle, ketone body synthesis, and gluconeogenesis. The contribution to these important processes could spare other ketogenic and gluconeogenic AA, which support the observed down‐regulation of glycine, serine, and threonine metabolism and biosynthesis of AA pathways.

### 4.3 Modulation of Protein Synthesis and Ribosomal Activity by BCAA and BCKA

The ribosome serves as the central hub for translational control, with its biogenesis and function being dynamically regulated by nutrient availability, particularly AA [33]. Our investigation of BCAA and BCKA supplementation reveals that both enhance ribosomal function and protein synthesis, though to differing degrees. BCAA supplementation increased the abundance of 22 ribosomal proteins, including RPL14, RPS3, RPL4, and RPS27L, demonstrating stimulated ribosome biogenesis and protein synthetic capacity. Supporting an increased protein synthesis, an abundance of 13 proteins in protein processing in endoplasmic reticulum were also upregulated, including Protein Disulfide Isomerase A3 (PDIA3) and Prolyl 4‐hydroxylase beta subunit (P4HB), which are typically upregulated during increased synthesis of secretory and structural proteins [34]. GO analysis further confirmed enrichment in translation (GO:0006412), protein folding (GO:0042026), and structural roles of ribosomal components (GO:0003735). Such extensive upregulation, occurring in parallel with enhanced expression of AA biosynthetic pathway components, indicates that hepatocytes preferentially utilize supplemented BCAA for anabolic processes.

BCKA supplementation similarly promoted ribosomal protein expression but with a more modest effect (8 proteins upregulated). This quantitative difference likely stems from two interrelated factors: (1) the high BCKD activity in hepatocytes directs BCKA primarily toward oxidative energy production, and (2) this metabolic contribution preserves other AA from catabolic breakdown, thereby increasing their availability for protein synthesis. Supporting this interpretation, we observed downregulation of glycine, serine, and threonine metabolic pathways—an indication of AA sparing.

Together, these findings reveal distinct but complementary mechanisms of ribosomal regulation: BCAA supplementation directly fuels anabolic processes through enhanced substrate availability, while BCKA supplementation indirectly supports protein synthesis by optimizing AA utilization through energy provision. Both pathways ultimately converge on enhanced translational capacity, albeit through different metabolic routes.

### 4.4 Distinct Impact on Energy Metabolism

Our findings reveal distinct metabolic adaptations in bovine hepatocytes following equimolar BCAA and BCKA supplementation under physiologically high FA conditions, particularly impacting the tricarboxylic acid (TCA) cycle, oxidative phosphorylation, and thermogenesis pathways. Specifically, BCAA supplementation upregulated these metabolic pathways, as evidenced by increased abundance of key enzymes, whereas BCKA supplementation elicited the opposite effect.

BCAA treatment significantly upregulated TCA cycle enzymes, including succinyl‐CoA ligase (SUCLG1), aconitase 2 (ACO2), malate dehydrogenase 2 (MDH2), isocitrate dehydrogenase 2 (IDH2), and malate dehydrogenase 1 (MDH1), suggesting enhanced flux through the TCA cycle and supporting increased energy production. Consistent with this, oxidative phosphorylation was also upregulated, marked by increased abundance of ATP synthase subunits (ATP5F1D, ATP5F1A, ATP5PB, ATP5F1B, ATP5F1C) and ubiquinol‐cytochrome c reductase (UQCRC2). These mitochondrial components indicate enhanced ATP production capacity in hepatocytes exposed to BCAA. Additionally, proteins associated with thermogenesis, including ATP synthase subunits and UQCRC2, exhibited upregulation, reinforcing the stimulatory role of BCAA on energy metabolism pathways [10, 35].

Conversely, BCKA supplementation downregulated these metabolic pathways. The abundance of key TCA cycle enzymes such as citrate synthase (CS), MDH2, phosphoenolpyruvate carboxykinase 2 (PCK2), and succinate dehydrogenase complex subunit B (SDHB) was decreased compared to CON. Similarly, oxidative phosphorylation was downregulated, with reduced abundance of ATP5F1B, NADH‐ubiquinone oxidoreductase subunit S3 (NDUFS3), SDHB, and cytochrome c oxidase subunit 5B (COX5B). Thermogenesis‐related proteins (e.g., NDUFS3, SDHB, COX5B) also exhibited reduced levels, indicating decreased mitochondrial energy production.

Given the high BCKD activity in hepatocytes, the observed downregulation of TCA cycle and oxidative phosphorylation enzymes upon BCKA supplementation contradicted our initial expectations. This unexpected finding may reflect a protective metabolic feedback mechanism preventing excessive reactive oxygen species (ROS) generation and metabolic stress triggered by BCKA metabolism. This hypothesis aligns with our previous in vivo findings demonstrating increased hepatic glutathione abundance and elevated circulating thiols in cows supplemented with BCKA [36]. Supporting this interpretation, Gene Ontology (GO) enrichment analysis revealed significant enrichment of oxidative stress response processes (GO:1902175, GO:0034599) in the BCAA versus BCKA comparison. Furthermore, the upregulation of oxidative phosphorylation and TCA cycle enzymes following BCAA supplementation appears congruent with enhanced FA oxidation, facilitating increased flux through the TCA cycle and ETC. Reinforcing this metabolic shift, pathway analysis revealed significant enrichment of FA metabolism pathways in the BCAA versus BCKA comparison, highlighted by the upregulated proteins very long‐chain specific acyl‐CoA dehydrogenase (ACADVL), sterol carrier protein 2 (SCP2), acetyl‐CoA acetyltransferase 1 (ACAT1), and fatty acid synthase (FASN).

## 5 Conclusions

This study demonstrates that supplementation with BCAA and BCKA reduces intracellular lipid droplet size and number in bovine hepatocytes when exposed to physiologically high FA. Despite equimolar supplementation, BCAA predominantly supports anabolic processes, enhancing amino acid biosynthesis, ribosomal biogenesis, and protein synthesis, whereas BCKA preferentially contributes to energy production, sparing other amino acids from catabolism.

Furthermore, BCAA supplementation enhanced hepatic energy metabolism, including the TCA cycle and oxidative phosphorylation, facilitating increased ATP production. Conversely, BCKA supplementation unexpectedly reduced these pathways, possibly reflecting a protective response against oxidative stress. These findings highlight the distinct yet complementary metabolic roles of BCAA and BCKA, offering insights into their therapeutic potential to mitigate hepatic lipid accumulation during critical physiological states in dairy cows.

## Author Contributions

Z.Z. and J.R.D. conceived and supervised the project. Z.Z., and J.R.D. designed the experiments. J.R.D. and M.S. conducted the experiments. J.R.D. performed the bioinformatics analysis. J.R.D. performed the confocal‐based quantifications. J.R.D., E.K., and M.G. prepared most of the figures and wrote the manuscript with help from all authors.

## Funding Information

This work is supported by the Agriculture and Food Research Initiative competitive grant no. 2021‐67015‐33383 from the USDA National Institute of Food and Agriculture (Washington, DC) and USDA, and AgBioResearch at Michigan State University.

## Conflicts of Interest

The authors have declared no conflicts of interest.

## Supporting information




**Supporting File**: pmic70132‐sup‐0001‐SuppMat.docx.

## Data Availability

The mass spectrometry proteomics data have been deposited to the ProteomeXchange Consortium via the PRIDE partner repository with the dataset identifier PXD062787 and are publicly accessible at: https://www.ebi.ac.uk/pride/archive/projects/PXD062787.

## References

[1] G. Bobe , J. W. Young , and D. C. Beitz , “Invited Review: Pathology, Etiology, Prevention, and Treatment of Fatty Liver in Dairy Cows,” Journal of Dairy Science 87 (2004): 3105–3124. 15377589 10.3168/jds.S0022-0302(04)73446-3

[2] C. B. Newgard , J. An , J. R. Bain , et al., “A Branched‐chain Amino Acid‐related Metabolic Signature That Differentiates Obese and Lean Humans and Contributes to Insulin Resistance,” Cell Metabolism 9 (2009): 311–326. 19356713 10.1016/j.cmet.2009.02.002 PMC3640280

[3] J. Rehage , C. Meier , M. Kaske , and H. Scholz , “Changes in the Amino Acid Ratio and Ammonia Concentration in Plasma and Cerebrospinal Fluid of Dairy Cows Suffering From Hepatosteatosis and Liver Failure,” Journal of Dairy Science 84 (2001): 152.

[4] M. Gaggini , F. Carli , C. Rosso , et al., “Altered Amino Acid Concentrations in NAFLD: Impact of Obesity and Insulin Resistance,” Hepatology 67 (2018): 210. 10.1002/hep.29465 28802074

[5] M. Charlton , “Branched‐chain Amino Acid Enriched Supplements as Therapy for Liver Disease,” Journal of Nutrition 136 (2006): 295S–298S. 16365102 10.1093/jn/136.1.295S

[6] M. Holeček , “Branched‐chain Amino Acids in Health and Disease: Metabolism, Alterations in Blood Plasma, and as Supplements,” Nutrition and Metabolism 15 (2018): 33. 29755574 10.1186/s12986-018-0271-1 PMC5934885

[7] R. J. Early , J. R. Thompson , R. J. Christopherson , and G. W. Sedgwick , “Blood Branched‐Chain Amino and α‐keto Acid Concentrations and Net Exchange Across the Portal‐Drained Viscera and Hindlimb of Fed and Fasted Ruminants,” Canadian Journal of Animal Science 67 (1987): 1011–1020.

[8] A. Suryawan , J. W. Hawes , R. A. Harris , Y. Shimomura , A. E. Jenkins , and S. M. Hutson , “A Molecular Model of human Branched‐Chain Amino Acid Metabolism,” American Journal of Clinical Nutrition 68 (1998): 72–81. 9665099 10.1093/ajcn/68.1.72

[9] Y. Zhang , L. Zhan , L. Zhang , Q. Shi , and L. Li , “Branched‐Chain Amino Acids in Liver Diseases: Complexity and Controversy,” Nutrients 16 (2024): 277. 38931228 10.3390/nu16121875 PMC11206364

[10] C. Nie , T. He , W. Zhang , G. Zhang , and X. Ma , “Branched Chain Amino Acids: beyond Nutrition Metabolism,” International Journal of Molecular Sciences 19 (2018): 954. 29570613 10.3390/ijms19040954 PMC5979320

[11] Y. Shimomura , T. Murakami , N. Nakai , M. Nagasaki , and R. A. Harris , “Exercise Promotes BCAA Catabolism: Effects of BCAA Supplementation on Skeletal Muscle During Exercise,” Journal of Nutrition 134 (2004): 1583S–1587S. 15173434 10.1093/jn/134.6.1583S

[12] J. R. Daddam , M. Sura , D. Vocelle , J. G. Laguna , K. Gallagher , and Z. Zhou , “The Supply of Branched‐chain Amino Acids and Branched‐Chain Keto Acids Alter Lipid Metabolism, Oxidative Stress, and Apoptosis in Primary Bovine Hepatocytes,” The Journal of Nutritional Biochemistry 137 (2025): 109839. 39805371 10.1016/j.jnutbio.2025.109839

[13] Y. F. Zhou , Z. Zhou , F. Batistel , et al., “Methionine and Choline Supply Alter Transmethylation, Transsulfuration, and Cytidine 5′‐diphosphocholine Pathways in Isolated Primary Liver Cells From Dairy Cows,” Journal of Dairy Science 101 (2018): 11384–11395. 30316602 10.3168/jds.2017-14236

[14] C. D. Tavares , K. Sharabi , J. E. Dominy , et al., “The Methionine Transamination Pathway Controls Hepatic Glucose Metabolism via GCN5 Acetyltransferase and PGC‐1α,” Journal of Biological Chemistry 291 (2016): 10635–10645. 27022023 10.1074/jbc.M115.706200 PMC4865912

[15] C. K. Reynolds , P. C. Aikman , B. Lupoli , D. J. Humphries , and D. E. Beever , “Splanchnic Metabolism of Dairy Cows From Late Gestation to Early Lactation,” Journal of Dairy Science 86 (2003): 1201–1217. 12741545 10.3168/jds.S0022-0302(03)73704-7

[16] Z. Zhou , O. Bulgari , M. Vailati‐Riboni , et al., “Rumen‐protected Methionine Improves Immunometabolic Status in Peripartal Dairy Cows,” Journal of Dairy Science 99 (2016): 8956–8969. 27592438 10.3168/jds.2016-10986

[17] Z. Zhou , M. Vailati‐Riboni , D. N. Luchini , and J. J. Loor , “Methionine and Choline Supply Alter Plasma Amino Acid and One‐Carbon Metabolism Profiles,” Nutrients 9 (2017): 10. 10.3390/nu9010010 PMC5295054 28036059

[18] K. Gallagher , I. Bernstein , C. Collings , et al., “Abomasal Infusion of Branched‐chain Amino Acids or Branched‐Chain Keto‐Acids Alters Lactation Performance and Liver Triglycerides,” Journal of Animal Science and Biotechnology 15 (2024): 13. 38281954 10.1186/s40104-023-00973-7 PMC10823655

[19] J. M. Pell , E. M. Caldarone , and E. N. Bergman , “Leucine and Alpha‐Ketoisocaproate Metabolism in Sheep,” Metabolism 35 (1986): 1005–1016. 3773720 10.1016/0026-0495(86)90036-3

[20] Z. Zhou , J. J. Loor , F. Piccioli‐Cappelli , F. Librandi , G. E. Lobley , and E. Trevisi , “Circulating Amino Acids in Peripartal Dairy Cows Vary With Liver Functionality Index,” Journal of Dairy Science 99 (2016): 2257–2267. 26778311 10.3168/jds.2015-9805

[21] M. Zajec , J. M. Kros , D. A. T. Dekker‐Nijholt , et al., “Identification of Blood–Brain Barrier‐Associated Proteins in the human Brain,” Journal of Proteome Research 20 (2020): 531–537. 33226812 10.1021/acs.jproteome.0c00551

[22] J. R. Daddam , M. Sura , E. Sarmikasoglou , et al., “Differences in Amino Acid and Fatty Acid Metabolism Contribute to Feed Efficiency Variability,” Journal of Dairy Science 108 (2025): 8367–8379. 40447084 10.3168/jds.2025-26468

[23] J. Cox and M. Mann , “MaxQuant Enables High Peptide Identification Rates and Proteome‐wide Quantification,” Nature Biotechnology 26 (2008): 1367–1372. 10.1038/nbt.1511 19029910

[24] K.‐C. Cho , D. J. Clark , M. Schnaubelt , et al., “Deep Proteomics Using Two‐Dimensional Data‐Independent Acquisition,” Analytical Chemistry 92 (2020): 4217–4225. 32058701 10.1021/acs.analchem.9b04418 PMC7255061

[25] Y. A. Ben Meir , J. R. Daddam , G. Kra , et al., “Proteomic Analysis of Adipose Tissue Reveals Proteins Associated With Feed Efficiency,” Scientific Reports 12 (2022): 9721. 35697844 10.1038/s41598-022-13964-x PMC9192684

[26] U. Klingmüller , A. Bauer , S. Bohl , et al., “Primary Mouse Hepatocytes for Systems Biology Approaches,” IET Systems Biology 153 (2006): 433–447. 10.1049/ip-syb:20050067 17186705

[27] F. He , “Bradford Protein Assay,” Bio‐Protocol 1 (2011): 45.

[28] P. K. Smith , R. I. Krohn , G. T. Hermanson , et al., “Measurement of Protein Using Bicinchoninic Acid,” Analytical Biochemistry 150 (1985): 76–85. 3843705 10.1016/0003-2697(85)90442-7

[29] U. K. Laemmli , “Cleavage of Structural Proteins During the Assembly of the Head of Bacteriophage T4,” Nature 227 (1970): 680–685. 5432063 10.1038/227680a0

[30] H. Towbin , T. Staehelin , and J. Gordon , “Electrophoretic Protein Transfer From Gels to Nitrocellulose,” Proceedings of the National Academy of Sciences 76 (1979): 4350–4354. 10.1073/pnas.76.9.4350 PMC411572 388439

[31] W. N. Burnette , ““Western Blotting”: Electrophoretic Transfer of Proteins and Detection on Nitrocellulose,” Analytical Biochemistry 112 (1981): 195–203. 6266278 10.1016/0003-2697(81)90281-5

[32] J. T. Brosnan and M. E. Brosnan , “Branched‐Chain Amino Acids: Enzyme and Substrate Regulation,” Journal of Nutrition 136 (2006): 207S–211S. 16365084 10.1093/jn/136.1.207S

[33] K. Thedieck and M. N. Hall , “Translational Control by Amino Acids and Energy,” in Handbook of Cell Signaling, 2nd ed. (2010), 2285–2293.

[34] L. Ellgaard and L. W. Ruddock , “The human Protein Disulphide Isomerase family,” EMBO Reports 6 (2005): 28–32. 15643448 10.1038/sj.embor.7400311 PMC1299221

[35] X. Sun and M. B. Zemel , “Leucine and Calcium Regulate Fat Metabolism in Adipocytes and Muscle Cells,” Lipids 42 (2007): 297–305. 17406924 10.1007/s11745-007-3029-5

[36] G. Ahmad , J. R. Daddam , E. Trevisi , et al., “Effects of Abomasal Branched‐Chain Amino Acid Infusion on Liver Function and Oxidative Stress,” Journal of Dairy Science 107 (2024): 9309–9321. 39004121 10.3168/jds.2024-24914

