# Supplementary material for: Branched‐Chain Amino and Keto Acids Reduce Hepatocyte Lipid Droplet Size and Number via Distinct Proteomic Pathways
Source: Proteomics. 2026 Apr 20;26(7):146–57. doi: 10.1002/pmic.70132 (PMC13327708; doi:10.1002/pmic.70132)
Supplement: Supplementary file 1 — Supporting File: pmic70132‐sup‐0001‐SuppMat.docx. [file PMIC-26--s001.docx]

**Supplemental Material**

Table S1. List of all differentially abundant proteins (DAP) in each treatment comparison.

|  | **BCAA vs CON** | | **BCKA vs CON** | | **BCAA vs BCKA** | |
| --- | --- | --- | --- | --- | --- | --- |
| **Protein ID** | **FDR** | **Fold change** | **FDR** | **Fold change** | **FDR** | **Fold change** |
| AARS1 | 0.0079 | 1.963474 | - | - | 0.0039 | 2.263034 |
| ACADVL | 0.00073 | 0.678072 | - | - | 0.0046 | 0.678072 |
| ACO2 | 0.047 | 0.485427 | - | - | 0.03 | 0.678072 |
| ACTB | < 0.00010 | 0.925999 | 0.019 | -0.32193 | - | - |
| ACTN4 | 0.0067 | 0.765535 | - | - | 0.0024 | 0.678072 |
| AHCY | 0.0019 | 1 | - | - | 0.0013 | 0.925999 |
| AK3 | 0.032 | 3.169925 | - | - | - | - |
| AKR1B1 | 0.015 | 1 | - | - | 0.00036 | 1.887525 |
| AKR7A2 | 0.013 | 2.405992 | - | - | 0.023 | 1.485427 |
| ALB | 0.00014 | -0.152 | - | - | - | - |
| ALDH7A1 | 0.00047 | -4.32193 | - | - | - | - |
| ANP32A | 0.014 | -0.51457 | - | - | - | - |
| ANXA1 | 0.0025 | 0.678072 | - | - | 0.0012 | 0.765535 |
| ANXA11 | 0.0045 | 2.608809 | - | - | 0.0044 | 2.432959 |
| ANXA2 | 0.0022 | 0.584963 | - | - | 0.00024 | 0.678072 |
| ANXA4 | 0.0003 | 1 | - | - | 0.00022 | 1.070389 |
| ANXA5 | 0.005 | 0.678072 | - | - | 0.0001 | 0.925999 |
| APEX1 | 0.043 | 2.485427 | - | - | 0.032 | 2.035624 |
| ARCN1 | 0.043 | 3.277985 | - | - | - | - |
| ARF1 | 0.0016 | 1.584963 | 0.0016 | 1.137504 | - | - |
| ASNS | 0.0067 | 3.153805 | - | - | 0.021 | 1.632268 |
| ATOX1 | 0.0097 | 1.722466 | - | - | 0.0021 | 2.378512 |
| ATP1A1 | 0.00033 | 1.432959 | - | - | - | - |
| ATP5F1A | 0.0015 | 0.847997 | - | - | 0.0002 | 1.070389 |
| ATP5F1B | 0.014 | 0.485427 | 0.00073 | -0.51457 | 0.00022 | 0.925999 |
| ATP5F1C | 0.0085 | 2.104337 | - | - | 0.0068 | 2.169925 |
| ATP5F1D | 0.019 | 1.485427 | - | - | 0.028 | 1.432959 |
| ATP5MG | 0.011 | -1.73697 | - | - | - | - |
| ATP5PB | 0.018 | 1.201634 | - | - | 0.012 | 1.137504 |
| BAX | 0.01 | 2.405992 | - | - | 0.0052 | 2.378512 |
| BCAP31 | 0.032 | 5.357552 | - | - | - | - |
| CA2 | 0.043 | 4.459432 | - | - | - | - |
| CALM | 0.032 | -1 | - | - | - | - |
| CAP1 | 0.025 | 1.925999 | - | - | 0.0076 | 2.510962 |
| CAPG | 0.00075 | 0.584963 | 0.0088 | -0.51457 | - | - |
| CCT2 | < 0.00010 | 1.963474 | - | - | - | - |
| CCT3 | 0.0057 | 1 | - | - | 0.022 | 0.765535 |
| CCT5 | 0.019 | 1.536053 | - | - | 0.011 | 1.678072 |
| CCT7 | 0.014 | 1.847997 | - | - | 0.0059 | 1.485427 |
| CCT8 | 0.0014 | 1.263034 | - | - | 0.0051 | 1.201634 |
| CD44 | 0.011 | 1.432959 | - | - | - | - |
| CDH17 | 0.003 | -3.32193 | 0.046 | -1.32193 | - | - |
| CDK1 | 0.038 | 2.981853 | - | - | 0.024 | 3.201634 |
| CFDP2 | 0.031 | 1.807355 | - | - | - | - |
| CLDN4 | 0.005 | -1.73697 | - | - | 0.003 | -1.73697 |
| CLTC | 0.0016 | 1.070389 | - | - | 0.0024 | 1.263034 |
| CNN2 | 0.015 | 0.584963 | - | - | 0.017 | 0.765535 |
| COX6C | 0.002 | 2.070389 | - | - | 0.00057 | 2.655352 |
| CTH | < 0.00010 | 2.137504 | - | - | 0.0062 | 1.584963 |
| CTSD | < 0.00010 | 4.247928 | - | - | 0.00015 | 1.847997 |
| CTSZ | 0.02 | 1.536053 | - | - | 0.024 | 1.378512 |
| CYCS | 0.012 | -2.32193 | - | - | 0.017 | -2.32193 |
| DBI | 0.019 | 1.070389 | - | - | 0.017 | 1 |
| DBNL | 0.0061 | 2.459432 | - | - | 0.005 | 2.378512 |
| DDAH1 | 0.0029 | 1.807355 | - | - | 0.00021 | 2.887525 |
| DDX17 | 0.011 | 4.906891 | 0.021 | 3.70044 | - | - |
| DIABLO | 0.0031 | 1.432959 | - | - | 0.0096 | 1.070389 |
| EEF1A1 | 0.0015 | 2.137504 | 0.017 | 1 | 0.0045 | 1.137504 |
| EEF1D | 0.023 | 3.321928 | - | - | 0.023 | 2.485427 |
| EEF1G | 0.00043 | 1.584963 | 0.00088 | 1.201634 | 0.044 | 0.378512 |
| EEF2 | 0.00012 | 1.678072 | 0.0064 | 0.485427 | 0.00024 | 1.137504 |
| EIF4A1 | 0.0012 | 1.137504 | - | - | 0.02 | 0.584963 |
| EIF4B | 0.0002 | 4.857981 | - | - | 0.0035 | 2 |
| EZR | 0.013 | 2.104337 | - | - | 0.031 | 1.263034 |
| FABP3 | < 0.00010 | 2 | 0.0043 | 1.201634 | 0.0018 | 0.765535 |
| FASN | 0.02 | 5.044394 | - | - | 0.03 | 2.459432 |
| FDPS | 0.049 | 1.485427 | - | - | 0.031 | 1.536053 |
| FGA | 0.0015 | 5.285402 | 0.031 | 5.459432 | - | - |
| FHL2 | 0.027 | 4.459432 | - | - | 0.023 | 3.153805 |
| FKBP1A | 0.0042 | 0.678072 | - | - | - | - |
| FLNA | 0.0042 | 0.765535 | - | - | 0.0052 | 0.678072 |
| FUBP1 | 0.032 | 2.169925 | - | - | - | - |
| GANAB | 0.037 | 1.070389 | - | - | 0.0037 | 1.485427 |
| GAPDH | < 0.00010 | 0.678072 | - | - | 0.0014 | 0.485427 |
| GATM | < 0.00010 | 1.321928 | - | - | 0.0006 | 0.765535 |
| GCLC | 0.043 | 1.963474 | 0.027 | 0.847997 | - | - |
| GLB1 | 0.002 | -3.8365 | 0.024 | -2.32193 | - | - |
| GNG2 | 0.015 | -2.32193 | - | - | 0.026 | -2.32193 |
| GOLGB1 | 0.043 | 2.722466 | - | - | 0.015 | 4.247928 |
| GOT2 | 0.049 | 0.485427 | - | - | 0.012 | 0.847997 |
| H1-3 | 0.047 | 2.104337 | - | - | - | - |
| H2AC19 | 0.029 | 1.632268 | 0.047 | 1.378512 | - | - |
| HADHA | 0.0041 | 1.584963 | - | - | 0.001 | 1.887525 |
| HDGF | 0.00097 | 1.678072 | - | - | 0.01 | 1.263034 |
| HINT2 | 0.002 | 2.378512 | - | - | 0.00034 | 3.321928 |
| HNRNPAB | 0.00012 | 2.744161 | - | - | - | - |
| HNRNPM | 0.015 | 1.887525 | 0.0087 | 1.137504 | - | - |
| HNRNPU | 0.032 | 2.104337 | - | - | - | - |
| HSP90AA1 | 0.0032 | 0.847997 | - | - | 0.02 | 0.584963 |
| HSP90AB1 | 0.00018 | 1.070389 | - | - | 0.0033 | 0.678072 |
| HSP90B1 | 0.00025 | 1 | - | - | 0.00028 | 0.925999 |
| HSPA1A | 0.0058 | 1.432959 | - | - | 0.012 | 1.137504 |
| HSPA4 | 0.024 | 0.847997 | - | - | 0.02 | 0.925999 |
| HSPA5 | 0.047 | 0.378512 | 0.036 | -0.32193 | 0.003 | 0.678072 |
| HSPA8 | 0.00026 | 1.070389 | - | - | 0.00049 | 0.847997 |
| HSPB1 | < 0.00010 | 1.137504 | 0.026 | -0.51457 | - | - |
| HSPD1 | 0.0085 | 0.925999 | - | - | 0.0016 | 1.137504 |
| IDH2 | < 0.00010 | 1.807355 | - | - | - | - |
| IGF2R | 0.011 | 5.906891 | 0.025 | 4 | 0.03 | 1.925999 |
| IQGAP1 | 0.0061 | 1.137504 | - | - | 0.0011 | 0.678072 |
| ITGA6 | 0.00069 | 4.321928 | - | - | 0.0012 | 2.632268 |
| KHSRP | 0.031 | 1.137504 | - | - | 0.05 | 1.321928 |
| KPNB1 | 0.0012 | 1 | - | - | 0.00012 | 1.485427 |
| KRT18 | 0.0015 | 0.678072 | - | - | 0.013 | 0.485427 |
| LAD1 | 0.0012 | 2.263034 | - | - | 0.0095 | 1.378512 |
| LASP1 | 0.0022 | 3.104337 | - | - | 0.0013 | 2.510962 |
| LDHA | < 0.00010 | 1.432959 | 0.045 | 0.584963 | 0.00076 | 0.847997 |
| LDHB | 0.00018 | 1.378512 | - | - | 0.0004 | 1 |
| LETM1 | 0.0026 | 2.459432 | - | - | 0.0037 | 1.847997 |
| LGALS1 | 0.0016 | -1 | 0.038 | -0.51457 | - | - |
| LGALS3 | 0.027 | 0.765535 | - | - | 0.0014 | 1.137504 |
| LMNA | 0.0011 | 1.321928 | - | - | 0.0015 | 1.137504 |
| LMNB1 | 0.0027 | 2.232661 | - | - | 0.0016 | 2.104337 |
| LYPLA1 | 0.027 | 1.432959 | - | - | - | - |
| MDH1 | 0.00025 | 1.485427 | - | - | 0.00018 | 1.536053 |
| MDH2 | 0.018 | 0.485427 | 0.035 | -0.51457 | 0.00032 | 1 |
| MT2A | 0.027 | 3.307429 | - | - | - | - |
| MTCH2 | 0.033 | 2.104337 | - | - | 0.038 | 1.536053 |
| MTHFD1 | 0.007 | 2.263034 | 0.014 | 1.722466 | - | - |
| MYL6 | 0.016 | 1.263034 | - | - | 0.025 | 1.070389 |
| NACA | 0.0027 | 1.201634 | - | - | - | - |
| NCL | < 0.00010 | 1.632268 | - | - | 0.00023 | 0.847997 |
| NPEPL1 | 0.00032 | 2.608809 | - | - | 0.00017 | 2.432959 |
| NPM1 | 0.0069 | 1.137504 | - | - | 0.014 | 0.925999 |
| ORM1 | 0.0098 | -1 | - | - | - | - |
| P4HB | 0.00062 | 0.925999 | 0.033 | 0.485427 | 0.012 | 0.485427 |
| PABPC1 | 0.013 | 1.378512 | - | - | - | - |
| PCBP1 | 0.00086 | 1 | - | - | 0.016 | 0.584963 |
| PDCD6IP | 0.042 | 2.263034 | - | - | - | - |
| PDIA3 | 0.0036 | 1.321928 | - | - | 0.005 | 0.925999 |
| PGK1 | 0.029 | 0.678072 | - | - | 0.0093 | 0.925999 |
| PGM2 | 0.017 | 2.137504 | - | - | 0.012 | 1.925999 |
| PHB | 0.046 | 0.678072 | 0.012 | -1.32193 | 0.0021 | 1.847997 |
| PHGDH | 0.001 | 0.584963 | - | - | 0.00047 | 0.678072 |
| PKM | 0.0095 | 0.485427 | - | - | 0.0023 | 0.678072 |
| PLEC | 0.031 | 1.201634 | - | - | - | - |
| PNP | 0.0037 | 0.765535 | - | - | 0.0048 | 0.678072 |
| PPIA | 0.0051 | 1.137504 | - | - | 0.0025 | 1.201634 |
| PPL | 0.00039 | 3.053111 | - | - | 0.0014 | 1.963474 |
| PRDX2 | 0.00096 | 1.070389 | - | - | 0.00018 | 1.321928 |
| PRDX5 | 0.037 | 0.678072 | 0.036 | 0.584963 | - | - |
| PSAP | 0.00093 | -3.47393 | - | - | 0.016 | -3.47393 |
| PSAT1 | 0.0024 | 0.925999 | - | - | 0.0033 | 0.847997 |
| PSMA5 | 0.046 | 1.432959 | - | - | - | - |
| PSMA7 | 0.0074 | -3.32193 | - | - | 0.00083 | -3.47393 |
| PSMB6 | 0.01 | 3.263034 | - | - | 0.0044 | 3.277985 |
| PSMC2 | 0.0017 | 2.485427 | - | - | - | - |
| PSMD2 | 0.021 | 5.087463 | - | - | 0.025 | 2.847997 |
| PSME2 | 0.0052 | -3.64386 | - | - | 0.0093 | -3.64386 |
| PTBP1 | 0.034 | 1.847997 | - | - | - | - |
| PTMA | 0.012 | 0.678072 | - | - | 0.034 | 0.584963 |
| PYCR1 | 0.044 | 1.847997 | - | - | 0.031 | 2.169925 |
| RAB5B | 0.0011 | 1.678072 | - | - | 0.0095 | 1.378512 |
| RAC1 | 0.00099 | 2.981853 | - | - | 0.00043 | 2.847997 |
| RPL10A | 0.024 | 3.906891 | - | - | - | - |
| RPL11 | 0.026 | 1.070389 | - | - | 0.018 | 0.925999 |
| RPL13 | 0.00094 | 1.632268 | - | - | 0.017 | 0.847997 |
| RPL14 | < 0.00010 | 1.722466 | - | - | 0.00033 | 1.321928 |
| RPL18 | 0.0022 | 4.954196 | 0.023 | 2.232661 | 0.0024 | 2.70044 |
| RPL18A | 0.0025 | 1.765535 | 0.0086 | 1.201634 | - | - |
| RPL28 | 0.023 | 4.087463 | 0.021 | 2.827819 | - | - |
| RPL30 | 0.022 | 1.263034 | - | - | 0.007 | 1.485427 |
| RPL34 | 0.0013 | 2.070389 | - | - | 0.024 | 1 |
| RPL37A | 0.012 | 4.643856 | - | - | 0.017 | 2.536053 |
| RPL4 | 0.034 | 1.847997 | - | - | 0.048 | 1.201634 |
| RPL7A | 0.0004 | 2.485427 | - | - | 0.0062 | 1.263034 |
| RPL8 | < 0.00010 | 2.201634 | 0.0087 | 1.201634 | 0.00071 | 1 |
| RPLP0 | 0.00064 | 1.137504 | - | - | - | - |
| RPN1 | 0.0094 | 5.209453 | - | - | - | - |
| RPS10 | 0.02 | 2.655352 | - | - | - | - |
| RPS11 | 0.00046 | 5.61471 | - | - | 0.0055 | 2.070389 |
| RPS13 | 0.00038 | 3.232661 | 0.042 | 2.263034 | 0.02 | 0.925999 |
| RPS14 | 0.0056 | 1.678072 | 0.022 | 1.070389 | - | - |
| RPS15A | < 0.00010 | 2.485427 | 0.022 | 2.070389 | - | - |
| RPS19 | 0.0051 | 2.632268 | 0.011 | 2.035624 | - | - |
| RPS21 | 0.0079 | 2.137504 | - | - | 0.019 | 1.536053 |
| RPS27A | 0.006 | -1.73697 | - | - | 0.01 | -1.32193 |
| RPS27L | 0.0099 | 2 | - | - | 0.037 | 1.201634 |
| RPS3 | 0.0016 | 1 | - | - | 0.047 | 0.378512 |
| RPS4Y1 | 0.02 | 2.378512 | - | - | - | - |
| RPS8 | < 0.00010 | 2 | - | - | - | - |
| RPSA | 0.023 | 1.137504 | - | - | 0.0073 | 1.321928 |
| RUVBL2 | 0.018 | 1.765535 | - | - | 0.0022 | 2.722466 |
| S100A10 | 0.00059 | 3.584963 | - | - | 0.00012 | 5.169925 |
| SCARB2 | 0.00021 | -3.8365 | - | - | 0.045 | -3.32193 |
| SDHB | < 0.00010 | -4.32193 | 0.0057 | -2.32193 | - | - |
| SERPINC1 | 0.009 | -2.32193 | - | - | 0.0081 | -2.32193 |
| SFPQ | 0.012 | 4.087463 | - | - | 0.04 | 1.678072 |
| SLC25A6 | 0.015 | 0.584963 | - | - | 0.021 | 0.584963 |
| SLC9A3R1 | 0.015 | 2.765535 | - | - | 0.01 | 2.632268 |
| SND1 | 0.046 | 2.232661 | - | - | - | - |
| SPTBN4 | 0.014 | -3.32193 | - | - | 0.044 | -3.32193 |
| SSB | 0.021 | 1.321928 | - | - | - | - |
| SUCLG1 | 0.0027 | 1.536053 | - | - | 0.00022 | 1.925999 |
| SWAP70 | 0.0015 | 2.350497 | - | - | 0.0017 | 1.847997 |
| SYNCRIP | 0.035 | 2.137504 | - | - | - | - |
| TAGLN2 | 0.00034 | 0.925999 | - | - | - | - |
| TALDO1 | 0.00022 | 1.678072 | - | - | 0.0042 | 1.137504 |
| TARS1 | 0.0094 | 1.485427 | - | - | 0.021 | 1.263034 |
| TCP1 | 0.0019 | 2.963474 | - | - | 0.0058 | 1.765535 |
| TKT | < 0.00010 | 1.378512 | - | - | - | - |
| TLN1 | 0.0054 | 3.053111 | - | - | 0.019 | 1.536053 |
| TMED10 | < 0.00010 | 1.963474 | - | - | 0.0004 | 1.378512 |
| TMSB10 | 0.0017 | 1.378512 | - | - | 0.00085 | 1.263034 |
| TMSB4 | 0.037 | 0.765535 | - | - | 0.0076 | 1.321928 |
| TPI1 | < 0.00010 | 1.070389 | - | - | - | - |
| TPM4 | 0.023 | 1.263034 | - | - | 0.012 | 1.321928 |
| TPT1 | 0.025 | 1.070389 | - | - | 0.0051 | 1.678072 |
| TTR | < 0.00010 | -3.64386 | - | - | - | - |
| TUBB5 | 0.003 | 0.847997 | - | - | 0.00044 | 1 |
| TXN | 0.015 | 0.584963 | - | - | 0.0029 | 0.765535 |
| UBA1 | 0.0016 | 1.321928 | - | - | 0.00031 | 1.536053 |
| UQCRC2 | 0.023 | 0.678072 | - | - | 0.00039 | 1.765535 |
| VCP | 0.016 | 0.765535 | - | - | - | - |
| VDAC2 | 0.013 | 0.765535 | - | - | 0.005 | 0.847997 |
| VIM | 0.00092 | 0.584963 | - | - | 0.0014 | 0.378512 |
| VSIG10L | 0.0078 | 1.963474 | - | - | 0.0014 | 3.070389 |
| YBX3 | 0.014 | -1.32193 | - | - | 0.028 | -1.73697 |
| YWHAZ | 0.002 | 1 | - | - | 0.0038 | 0.765535 |
| ACAT1 | - | - | 0.043 | -0.73697 | 0.0072 | 1.070389 |
| ACTR3 | - | - | 0.013 | 2 | - | - |
| ATP1B1 | - | - | 0.017 | -3.32193 | - | - |
| COX5B | - | - | 0.04 | -1.32193 | - | - |
| CS | - | - | 0.049 | -0.73697 | 0.0089 | 1.137504 |
| GAPDHS | - | - | 0.029 | -1 | 0.0038 | 1.321928 |
| GBA | - | - | 0.0038 | -2.32193 | - | - |
| HNRNPA2B1 | - | - | 0.018 | 0.678072 | - | - |
| HNRNPA3 | - | - | 0.035 | 1.807355 | 0.013 | -2.32193 |
| HSPA9 | - | - | 0.0013 | -0.73697 | 0.00037 | 0.765535 |
| HSPE1 | - | - | 0.026 | -0.51457 | 0.0039 | 1 |
| LCP1 | - | - | 0.046 | -1.73697 | 0.0063 | 2.867896 |
| ME1 | - | - | 0.021 | -1 | - | - |
| NDUFS3 | - | - | 0.048 | -1.32193 | - | - |
| PA2G4 | - | - | 0.037 | 2.560715 | - | - |
| PCK2 | - | - | 0.033 | -0.51457 | - | - |
| PEBP1 | - | - | 0.026 | -0.73697 | 0.0054 | 1.321928 |
| PSMA3 | - | - | 0.032 | -2.32193 | 0.015 | 2.887525 |
| PSPH | - | - | 0.0043 | -1.32193 | 0.021 | 1.263034 |
| RPLP1 | - | - | 0.014 | 2.963474 | - | - |
| RPS12 | - | - | 0.021 | 0.584963 | - | - |
| SERPINH1 | - | - | 0.041 | -1.73697 | - | - |
| SHMT2 | - | - | 0.0075 | -1 | - | - |
| SQOR | - | - | 0.021 | -1.32193 | - | - |
| SULT1A1 | - | - | 0.012 | 1.485427 | - | - |
| VAT1 | - | - | 0.017 | -3.32193 | - | - |
| VDAC1 | - | - | 0.019 | -0.73697 | - | - |
| MYO1D | - | - | - | - | 0.036 | 4.321928 |
| PEA15 | - | - | - | - | 0.018 | 3.70044 |
| SNRPD3 | - | - | - | - | 0.0077 | 3.584963 |
| PSMD13 | - | - | - | - | 0.043 | 3.307429 |
| DCTN1 | - | - | - | - | 0.024 | 3.217231 |
| GSTA1 | - | - | - | - | 0.028 | 3.121015 |
| DYNC1H1 | - | - | - | - | 0.02 | 2.847997 |
| CSRP1 | - | - | - | - | 0.023 | 2.847997 |
| NDUFS1 | - | - | - | - | 0.0089 | 2.807355 |
| PAK2 | - | - | - | - | 0.011 | 2.678072 |
| KIF5B | - | - | - | - | 0.042 | 2.655352 |
| SARS1 | - | - | - | - | 0.00076 | 2.632268 |
| SSR4 | - | - | - | - | 0.01 | 2.432959 |
| SCP2 | - | - | - | - | 0.02 | 2.405992 |
| ATP5PO | - | - | - | - | 0.0029 | 2.321928 |
| ANXA3 | - | - | - | - | 0.02 | 2.169925 |
| CTSL | - | - | - | - | 0.0037 | 2 |
| ACO1 | - | - | - | - | 0.022 | 2 |
| WDR1 | - | - | - | - | 0.038 | 2 |
| ESD | - | - | - | - | 0.05 | 1.925999 |
| ZYX | - | - | - | - | 0.0097 | 1.807355 |
| PLS3 | - | - | - | - | 0.05 | 1.765535 |
| ABHD14B | - | - | - | - | 0.019 | 1.722466 |
| PRDX4 | - | - | - | - | 0.0071 | 1.584963 |
| SFXN1 | - | - | - | - | 0.04 | 1.584963 |
| S100A11 | - | - | - | - | 0.017 | 1.536053 |
| HNRNPH1 | - | - | - | - | 0.018 | 1.536053 |
| EIF5A | - | - | - | - | 0.046 | 1.536053 |
| RPL24 | - | - | - | - | 0.033 | 1.485427 |
| DPP3 | - | - | - | - | 0.042 | 1.485427 |
| SUCLG2 | - | - | - | - | 0.022 | 1.432959 |
| PHB2 | - | - | - | - | 0.00032 | 1.263034 |
| IDH3A | - | - | - | - | 0.031 | 1.263034 |
| CCT4 | - | - | - | - | 0.025 | 1.201634 |
| RNH1 | - | - | - | - | 0.037 | 1.070389 |
| PPIB | - | - | - | - | 0.042 | 1.070389 |
| CLIC1 | - | - | - | - | 0.014 | 1 |
| PARK7 | - | - | - | - | 0.024 | 1 |
| MTPN | - | - | - | - | 0.028 | 1 |
| ALDOA | - | - | - | - | 0.045 | 1 |
| GSTP1 | - | - | - | - | 0.00068 | 0.925999 |
| GARS1 | - | - | - | - | 0.005 | 0.925999 |
| CFL2 | - | - | - | - | 0.017 | 0.925999 |
| GDI2 | - | - | - | - | 0.028 | 0.847997 |
| GLUD1 | - | - | - | - | 0.017 | 0.765535 |
| INPP5E | - | - | - | - | 0.021 | 0.678072 |
| TUBA1D | - | - | - | - | 0.046 | 0.678072 |
| AHNAK | - | - | - | - | 0.033 | 0.485427 |
| S100A2 | - | - | - | - | 0.022 | 0.378512 |
| RTN4 | - | - | - | - | 0.043 | -0.51457 |
| NAP1L1 | - | - | - | - | 0.0016 | -1.73697 |
| RBMX | - | - | - | - | 0.023 | -3.32193 |
| SERBP1 | - | - | - | - | 0.034 | -4.05889 |

**Supplementary Figures**


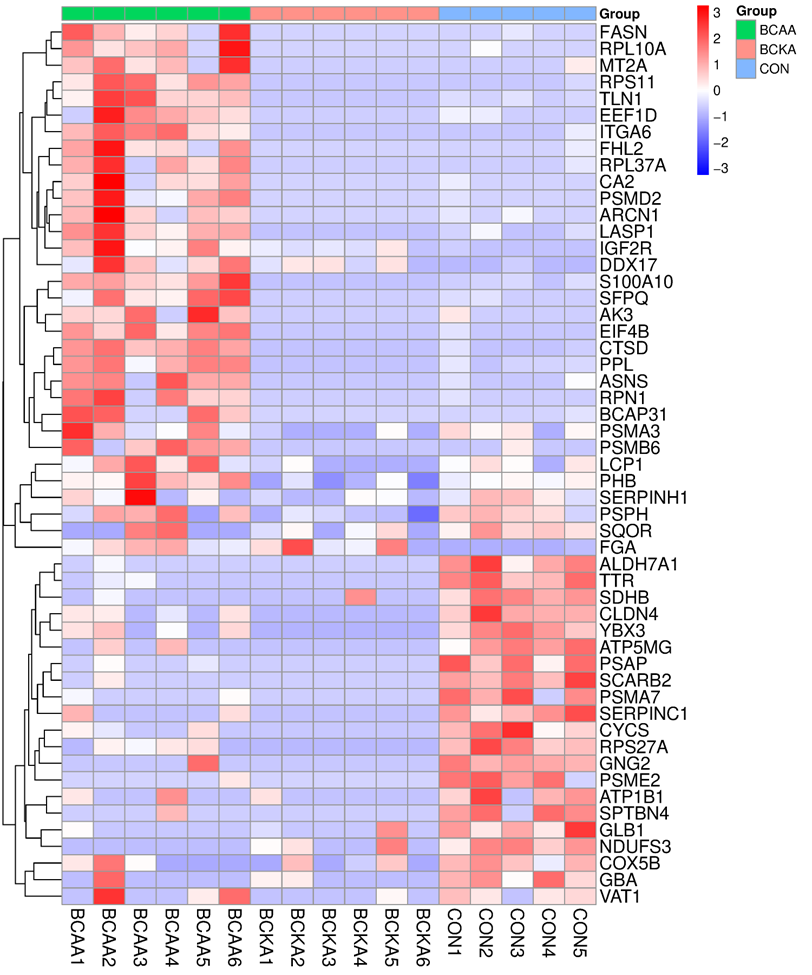


**Figure S1**: **Heat map visualization of differentially abundant proteins (DAPs) in hepatocytes treated with BCAA, BCKA, or control (CON) under high fatty acid conditions.** Hierarchical clustering was performed based on normalized protein abundance (Z-scores) across biological replicates (n = 6/group). Distinct proteomic profiles are evident among treatment groups, with clustering patterns reflecting treatment-specific regulation of metabolic and cellular pathways.


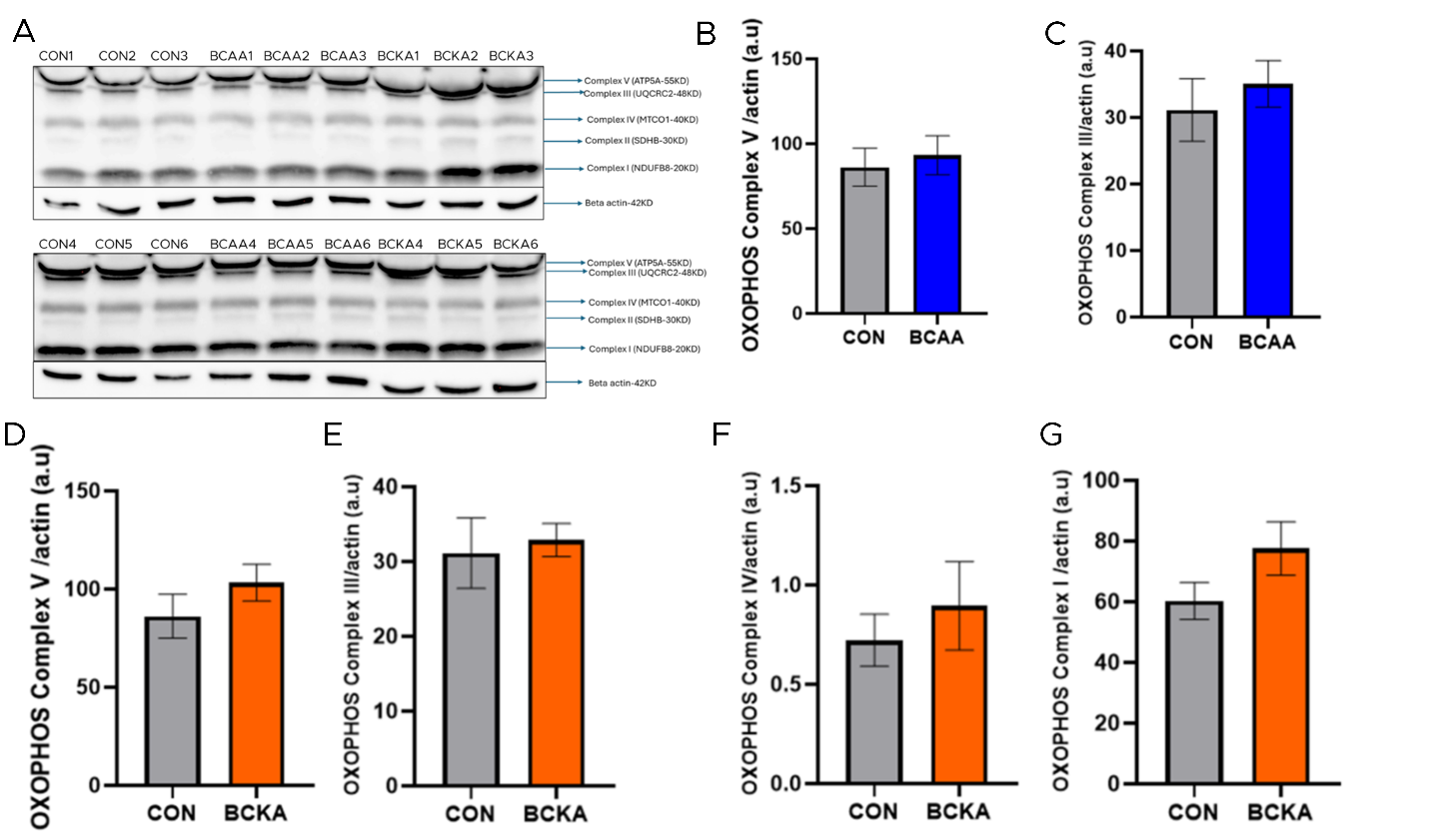


**Figure S2**: **Mitochondrial electron transport chain (ETC) complex abundance in hepatocytes receiving CON, BCAA or BCKA treatments.** (A) Hepatocytes were treated with BCAAs or BCKAs for 72 h, and the abundance of ETC complex proteins—ATP5A (Complex V), MTCO1 (Complex IV), UQCRC2 (Complex III), SDHB (Complex II), and NDUFB8 (Complex I)—was assessed by immunoblotting. Protein levels were normalized to β-actin. (B–C) Abundance of ATP5A (Complex V; B) and UQCRC2 (Complex III; C) in BCAA-treated hepatocytes relative to controls. (D–G) Abundance of ATP5A (D), UQCRC2 (E), MTCO1 (F), and NDUFB8 (G) in BCKA-treated hepatocytes relative to controls. β-actin served as a loading control.
